# Mechanisms contributing to hypotension after anesthetic induction with sufentanil, propofol, and rocuronium: a prospective observational study

**DOI:** 10.1007/s10877-021-00653-9

**Published:** 2021-02-01

**Authors:** Bernd Saugel, Elisa-Johanna Bebert, Luisa Briesenick, Phillip Hoppe, Gillis Greiwe, Dongsheng Yang, Chao Ma, Edward J. Mascha, Daniel I. Sessler, Dorothea E. Rogge

**Affiliations:** 1 Department of Anesthesiology, Center of Anesthesiology and Intensive Care Medicine, University Medical Center Hamburg-Eppendorf, Martinistrasse 52, 20246 Hamburg, Germany; 2 Outcomes Research Consortium, Cleveland, OH USA; 3 Departments of Quantitative Health Sciences and Outcomes Research, Lerner Research Institute and Anesthesiology Institute, Cleveland Clinic, Cleveland, OH USA; 4 Department of Outcomes Research, Anesthesiology Institute, Cleveland Clinic, Cleveland, OH USA

**Keywords:** Intraoperative hypotension, Blood pressure, Cardiac output, Hemodynamic monitoring, Cardiovascular dynamics

## Abstract

It remains unclear whether reduced myocardial contractility, venous dilation with decreased venous return, or arterial dilation with reduced systemic vascular resistance contribute most to hypotension after induction of general anesthesia. We sought to assess the relative contribution of various hemodynamic mechanisms to hypotension after induction of general anesthesia with sufentanil, propofol, and rocuronium. In this prospective observational study, we continuously recorded hemodynamic variables during anesthetic induction using a finger-cuff method in 92 non-cardiac surgery patients. After sufentanil administration, there was no clinically important change in arterial pressure, but heart rate increased from baseline by 11 (99.89% confidence interval: 7 to 16) bpm (P < 0.001). After administration of propofol, mean arterial pressure decreased by 23 (17 to 28) mmHg and systemic vascular resistance index decreased by 565 (419 to 712) dyn*s*cm^−5^*m^2^ (P values < 0.001). Mean arterial pressure was < 65 mmHg in 27 patients (29%). After propofol administration, heart rate returned to baseline, and stroke volume index and cardiac index remained stable. After tracheal intubation, there were no clinically important differences compared to baseline in heart rate, stroke volume index, and cardiac index, but arterial pressure and systemic vascular resistance index remained markedly decreased. Anesthetic induction with sufentanil, propofol, and rocuronium reduced arterial pressure and systemic vascular resistance index. Heart rate, stroke volume index, and cardiac index remained stable. Post-induction hypotension therefore appears to result from arterial dilation with reduced systemic vascular resistance rather than venous dilation or reduced myocardial contractility.

**Supplementary Information:**

The online version of this article (10.1007/s10877-021-00653-9) contains supplementary material, which is available to authorized users.

## Introduction

Intraoperative hypotension is associated with myocardial injury, acute kidney injury, and death [1–7]. The harm threshold appears to be a mean arterial pressure of about 65 mmHg, with risk progressively increasing at lower pressures and longer durations [8]. About a third of all intraoperative hypotension occurs between anesthetic induction and surgical incision [9, 10]. Since surgery has yet to start when this post-induction hypotension occurs, it is largely determined by patients’ baseline risk and anesthetic management [10, 11]—with the latter being modifiable.

Anesthesia is often induced with a combination of sufentanil, propofol, and rocuronium. The neuromuscular blocking agent rocuronium probably has little effect on arterial pressure besides hemodynamic effects related to paralysis itself [12]. However, opioids promote post-induction hypotension [11, 13], as does propofol [14–17]. It remains unclear, though, whether post-induction hypotension is primarily due to reduced myocardial contractility, venous dilation with decreased venous return, or arterial dilation with reduced systemic vascular resistance [18–20]. The relative contribution of different potential pathophysiologic mechanisms to hypotension after anesthetic induction thus remain unclear.

A better understanding of pathophysiologic mechanisms contributing to post-induction hypotension may guide management and reduce hypotension. We therefore sought to assess the relative contribution of various hemodynamic mechanisms to hypotension after induction of general anesthesia with sufentanil, propofol, and rocuronium in adults having non-cardiac surgery.

## Methods

### Study design

This was a prospective observational study performed in the Department of Anesthesiology, Center of Anesthesiology and Intensive Care Medicine, University Medical Center Hamburg-Eppendorf, Hamburg, Germany between April and August 2018. The study was approved by the Ethics Committee of the Medical Association of Hamburg on January 9, 2018. All patients provided written informed consent. This observational study adheres to the STROBE guidelines.

### Inclusion and exclusion criteria

We included adults with American Society of Anesthesiologists (ASA) physical status class I-III scheduled for elective gynecologic, urologic, otolaryngologic, or oral and maxillofacial surgery with general anesthesia and tracheal intubation. Patients were excluded if they had heart failure (New York Heart Association Functional Classification class II or higher), atrial fibrillation or other high-grade cardiac arrhythmias, peripheral artery occlusive disease (Fontaine stage II or higher), took beta blockers, had edema of the hands or fingers, had a history or suspicion of difficult airway, or an indication for rapid sequence induction. Patients were also excluded when regional anesthesia was performed before induction of anesthesia.

### Study protocol and measurements

Patients were not premedicated. Preoxygenation was performed with a sealed face mask at a positive end-expiratory pressure of 5 mbar. Anesthesia was induced with sufentanil (0.2–0.5 µg*kg^−1^), propofol (1.5–2.5 mg*kg^−1^), and rocuronium (0.5–0.9 mg*kg^−1^). Patients' tracheas were intubated and mechanical ventilation was initiated with a tidal volume of 6–8 mL*kg^−1^ at a positive end-expiratory pressure of 5 mbar. After induction, general anesthesia was maintained with either propofol or inhaled sevoflurane.

In addition to routine anesthetic monitoring, we continuously measured hemodynamic variables using a non-invasive finger-cuff method (CNAP; CNSystems Medizintechnik GmbH, Graz, Austria). The CNAP system was calibrated to brachial arterial pressure obtained from the system's upper-arm cuff. The CNAP system provides continuous arterial pressure values and waveforms. Using pulse wave analysis, the CNAP system also estimates advanced hemodynamic variables including cardiac output and systemic vascular resistance. The CNAP system was validated in several clinical studies showing that it reliably estimates arterial pressure and cardiac output [21–25].

**Fig. 1:**
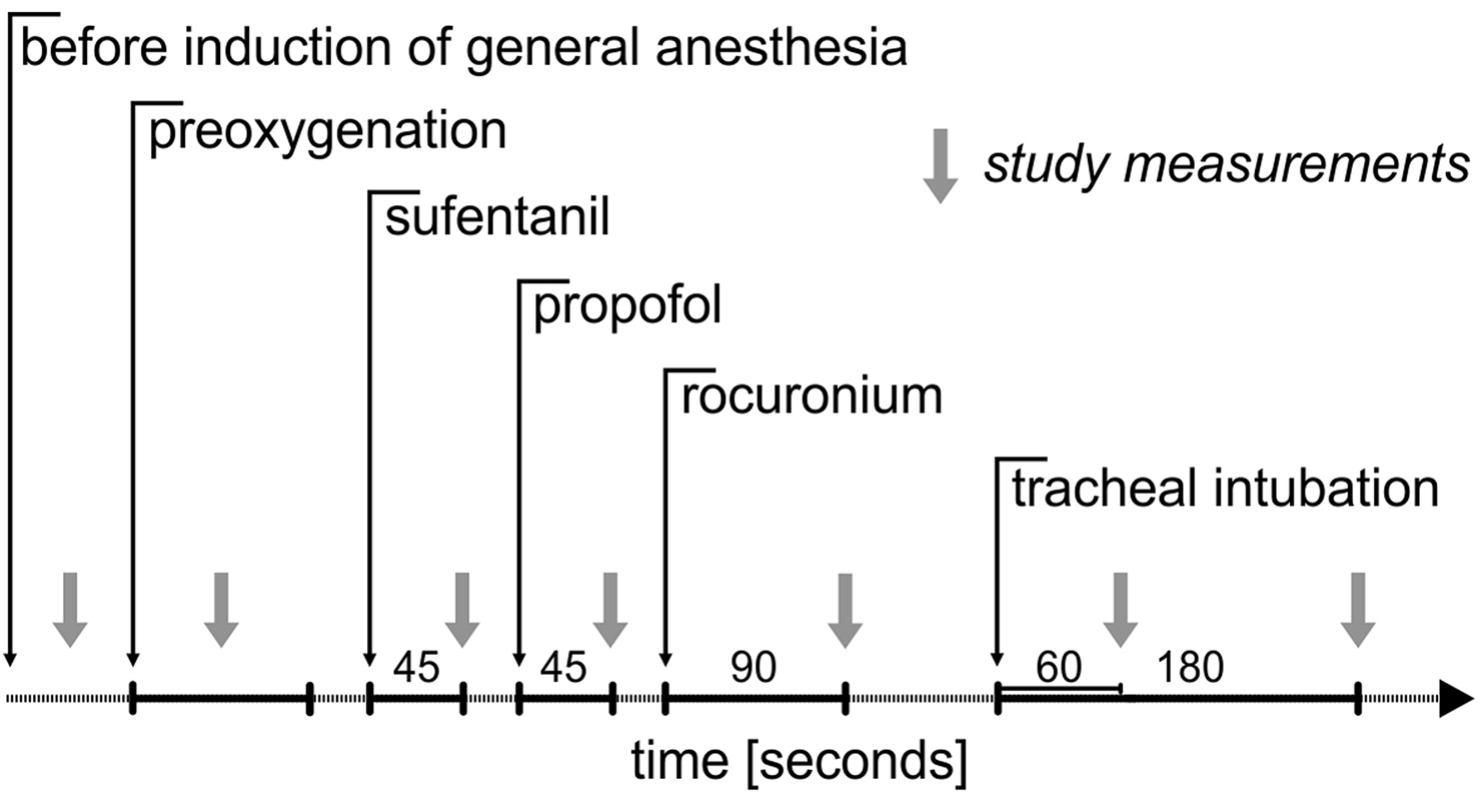
Measurement time points. We recorded hemodynamic variables before induction of general anesthesia, during preoxygenation, 45 s after administration of sufentanil, 45 s after administration of propofol, 90 s after administration of rocuronium, 60 s after tracheal intubation, and 180 s after tracheal intubation

We recorded arterial pressure, heart rate, cardiac index, stroke volume index, and systemic vascular resistance index at the following time points (Fig. 1): before induction of general anesthesia, during preoxygenation, 45 s after administration of sufentanil, 45 s after administration of propofol, 90 s after administration of rocuronium, 60 s after tracheal intubation, and 180 s after tracheal intubation.

### Statistical analysis

Data are presented as mean ± standard deviation (SD) for continuous variables and n (%) for categorical variables. Linear mixed effects models were used to estimate change from baseline (i.e., before induction of anesthesia) in various hemodynamic variables to 6 time points during induction using an autoregressive (AR (1)) covariance structure. The overall significance level was 0.05; Bonferroni correction was used to control the type I error for 7 outcomes and 6 comparisons within each outcome, and the significant level for each comparison was 0.0011 (i.e., alpha = 0.05/7/6 = 0.0011).

## Results

We enrolled 125 patients but excluded 28 who were given norepinephrine (14 patients) or additional doses of propofol (14 patients) during the study period. We also excluded 5 patients because of technical problems during data recording. We thus included 92 patients in the final analysis.

Participating patients were young, with a mean ± SD age of 36 ± 13 years and relatively healthy with 91% having ASA physical status class I or II (Table 1). General anesthesia was induced with 35 ± 6 µg of sufentanil, 187 ± 39 mg of propofol, and 37 ± 8 mg of rocuronium.

**Table 1:** Baseline characteristics

Factor	n_missing_	Total (n = 92)
Demographic		
Age, years	0	36 ± 13
Female, n (%)	0	55 (60)
Height, cm	0	172 ± 9
Weight, kg	0	74 ± 17
Body mass index, kg*m^-^^2^	0	25 ± 5
ASA physical status class, n (%)	0	
1		35 (38)
2		49 (53)
3		8 (9)
Induction medication		
Sufentanil (µg)	0	35 ± 6
Propofol (mg)	0	187 ± 39
Rocuronium (mg)	0	37 ± 8

Statistics presented as mean ± standard deviation

*ASA*, American Society of Anesthesiologists

**Fig. 2:**
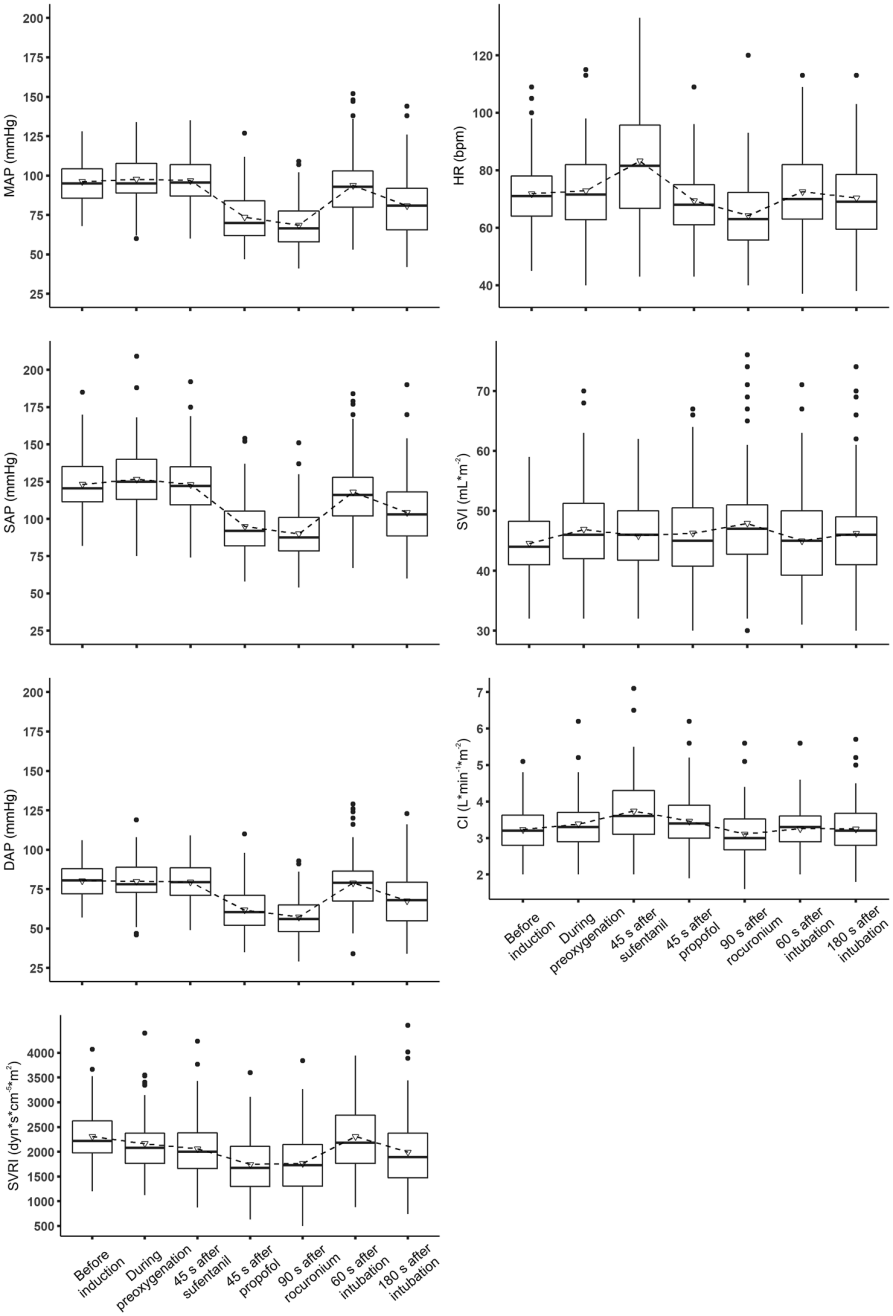
Hemodynamic variables during the induction of general anesthesia. Boxplots showing mean (triangle) and median (horizontal bar) with 25th–75th percentile (box) of hemodynamic variables during the induction of general anesthesia. Whiskers extend to the most extreme observations within 1.5 times the interquartile range of the first and third quartiles, respectively. Circles represent outliers. *MAP* mean arterial pressure, *SAP* systolic arterial pressure, *DAP* diastolic arterial pressure, *SVRI* systemic vascular resistance index, *HR* heart rate, *SVI* stroke volume index, *CI* cardiac index

Hemodynamic variables at specified time points are shown in Fig. 2 and Supplemental Table S1. Patients were normotensive at baseline with mean arterial pressure being 96 ± 13 mmHg. At baseline, heart rate was 72 ± 13 bpm, cardiac index was 3.2 ± 0.6 L*min^−1^*m^−2^, stroke volume index was 45 ± 6 mL*m^−2^, and systemic vascular resistance index was 2309 ± 544 dyn*s*cm^−5^*m^2^.

After sufentanil administration, heart rate increased from baseline by 11 (99.89% confidence interval: 7 to 16) bpm (P < 0.001). As there was no clinically important change in stroke volume index after sufentanil administration the increase in heart rate resulted in a slight increase in cardiac index of 0.5 (0.3 to 0.7) L*min^−1^m^−2^. There was no clinically important change in arterial pressure after sufentanil administration.

After administration of propofol, mean arterial pressure decreased by 23 (17 to 28) mmHg and systemic vascular resistance index decreased by 565 (419 to 712) dyn*s*cm^-5^*m^2^ (P values < 0.001). After propofol administration, mean arterial pressure was < 65 mmHg in 27 patients (29%). Heart rate returned to baseline after administration of propofol, and stroke volume index and cardiac index remained stable compared to baseline.

After administration of rocuronium, mean arterial pressure, systemic vascular resistance index, and heart rate all were below baseline values (P values < 0.001), but transiently increased to baseline levels after tracheal intubation.

180 s after tracheal intubation, there were no clinically important differences compared to baseline in heart rate, stroke volume index, or cardiac index. However, arterial pressure and systemic vascular resistance index remained well below baseline. 180 s after tracheal intubation, mean arterial pressure was 15 (10 to 20) mmHg lower than at baseline and it was < 65 mmHg in 21 patients (23%).

Supplemental Figure S1 shows Spaghetti plots for individual patients and Supplemental Figure S2 shows boxplots of changes in hemodynamic variables over time.

## Discussion

In this prospective observational study, we sought to assess the relative contribution of various hemodynamic mechanisms to hypotension after induction of general anesthesia with sufentanil, propofol, and rocuronium in adults having non-cardiac surgery.

Heart rate and cardiac index increased after sufentanil administration, but presumably not due to a pharmacological effect of sufentanil. Instead, the increases likely reflect stress-induced sympathetic activation in anticipation of anesthetic induction. Propofol caused a clinically important reduction in arterial pressure. In addition, systemic vascular resistance index decreased significantly, by about 25%, after propofol administration. Heart rate returned to baseline after administration of propofol, and stroke volume index and cardiac index remained stable compared to baseline. Hypotension after propofol administration thus was linked to a decrease in systemic vascular resistance. Rocuronium administration had no additional clinically relevant effect on cardiovascular dynamics.

A controversy remains about propofol-induced post-induction hypotension. The main mechanisms proposed are a decrease in myocardial contractility, venous dilation with a decrease in venous return, and arterial dilation with a decrease in systemic vascular resistance [18–20]. Experimental and animal studies suggest that propofol reduces myocardial contractility. For example, propofol directly depresses myocardial contractility in isolated guinea pig myocardial trabeculae [26] and isolated perfused guinea pig hearts [27]. Propofol similarly reduces myocardial contractility in anesthetized rabbits [28]. Propofol decreases inotropy in anesthetized dogs, but also reduces arterial and venous vascular tone [29]. In 23 major abdominal surgery patients, propofol markedly decreased mean arterial pressure, heart rate, and cardiac output [17]. We found that neither stroke volume index nor cardiac index were reduced after propofol administration, suggesting that myocardial contractility was hardly influenced. Venous dilation has been proposed as a cause of propofol-induced hypotension [19, 30]. Venous dilation alone would reduce venous return to the heart, causing stroke volume to decrease. Since we did not observe a significant decrease in stroke volume index, propofol-induced venous dilation in our study seems unlikely. Our results thus suggest that propofol-induced post-induction hypotension results from arterial dilation with reduced systemic vascular resistance rather than venous dilation or reduced myocardial contractility. Our results are consistent with a previous small study which also reported decreased afterload without a compensatory increase in heart rate or cardiac output resulting in hypotension [31].

About a third of our patients had mean arterial pressures < 65 mmHg after propofol administration. While there is strong evidence that intraoperative hypotension is associated with postoperative organ failure and death [1–7] research only recently focused on characterizing different phases of intraoperative hypotension [9, 10]. For anesthesiologists it is crucial to acknowledge that about a third of all intraoperative hypotension occurs between anesthetic induction and surgical incision and that hypotension during this period appears equally harmful as hypotension that occurs during surgery [9]. Because post-induction hypotension is consequent to anesthetic drugs, much of it is presumably preventable—and probably should be prevented.

This reinforces the need to mitigate the potential cardiovascular effects of induction of general anesthesia. Our results indicate that post-induction hypotension results largely from arterial dilation, and therefore that vasopressors will generally be the most appropriate treatment. Which vasopressor(s) might be best remains unclear as there are sparse data related to the treatment or prophylaxis of post-induction hypotension by using vasopressors. In a preliminary study, phenylephrine and norepinephrine boluses effectively counteracted intraoperative hypotension caused by propofol anesthesia [32]. Although logic suggests that fluid loading may help prevent hypotension, pre-induction crystalloid loading does not prevent post-induction hypotension [33, 34]. Colloid loading may somewhat be more effective, but still fails to prevent much hypotension [35]. Vasopressors thus appear to be a preferable clinical strategy.

In our study, induction agents were standardized, but exact doses were not and remained at the discretion of the attending anesthesiologist. Additionally, we used a non-invasive finger-cuff method to assess advanced hemodynamic variables. The non-invasive monitoring system we used is well validated for the measurement of continuous blood pressure [21–23] and cardiac output [24, 25]. It is therefore unlikely that our overall conclusions would differ with invasive measurements. Further, we did not use echocardiography that could have provided important information on myocardial function. Our study was restricted to relatively young healthy adults and may thus not be generalizable to older and sicker patients, especially patients with cardiovascular co-morbidities.

## Conclusions

In patients having non-cardiac surgery, anesthetic induction with sufentanil, propofol, and rocuronium was associated with a clinically important (and statistically significant) reduction in arterial pressure and systemic vascular resistance index. Heart rate and stroke volume index, and therefore cardiac index, basically remained stable during anesthetic induction. Post-induction hypotension therefore appears to result from arterial dilation with reduced systemic vascular resistance rather than venous dilation or reduced myocardial contractility. Future research should evaluate strategies for early detection and avoidance of post-induction hypotension, especially the (preemptive) use of vasopressors.

## Electronic supplementary material

Below is the link to the electronic supplementary material.

Hemodynamic variables during the induction of general anesthesia in individual patients. Spaghetti plots are showing the changes in hemodynamic variables during the induction of general anesthesia in individual patients. Black and blue lines therefore show individual trajectories of hemodynamic variables over time. MAP, mean arterial pressure; SAP, systolic arterial pressure; DAP, diastolic arterial pressure; SVRI, systemic vascular resistance index; HR, heart rate; SVI, stroke volume index; CI, cardiac index. Supplementary material 1 (TIF 32885 kb)

Hemodynamic variables during the induction of general anesthesia (change from baseline). Boxplots showing the change of hemodynamic variables during the induction of general anesthesia compared to baseline. Boxplots show mean (triangle) and median (horizontal bar) with 25th-75th percentile (box). Whiskers extend to the most extreme observations within 1.5 times the interquartile range of the first and third quartiles, respectively. Circles represent outliers. MAP, mean arterial pressure; SAP, systolic arterial pressure; DAP, diastolic arterial pressure; SVRI, systemic vascular resistance index; HR, heart rate; SVI, stroke volume index; CI, cardiac index. Supplementary material 2 (TIF 12696 kb)

Supplementary material 3 (PDF 74 kb)

## Data Availability

Department of Anesthesiology, Center of Anesthesiology and Intensive Care Medicine, University Medical Center Hamburg-Eppendorf, Martinistrasse 52, 20246 Hamburg, Germany.
