# Supplementary material for: Mechanisms contributing to hypotension after anesthetic induction with sufentanil, propofol, and rocuronium: a prospective observational study
Source: J Clin Monit Comput. 2021 Feb 1;36(2):341–7. doi: 10.1007/s10877-021-00653-9 (PMC9122881; doi:10.1007/s10877-021-00653-9)
Supplement: Supplementary file 3 — Supplementary material 3 (PDF 74 kb) [file 10877_2021_653_MOESM3_ESM.pdf]

Supplemental Table S1: Summary of hemodynamic variables over time				
**Outcome**	**n missing**	**Mean ± standard deviation (n = 92)**	**Change from baseline (99.89% confidence interval)***	**P value***
**Mean arterial pressure, mmHg**				
Before start of induction		96 ± 13		
During preoxygenation with face mask		98 ± 15	2 (-4, 7)	0.24
45 s after sufentanil		97 ± 15	1 (-4, 6)	0.69
45 s after propofol		74 ± 15	-23 (-28, -17)	<0.001**
90 s after rocuronium		67 ± 15	-28 (-33, -23)	<0.001**
60 s after intubation	1	94 ± 21	-3 (-8, 3)	0.22
180 s after intubation	1	81 ± 20	-15 (-20, -10)	<0.001**
**Systolic blood pressure, mmHg**				
Before start of induction		123 ± 18		
During preoxygenation with face mask		127 ± 22	4 (-3, 10)	0.026
45 s after sufentanil		123 ± 21	-0.1 (-6, 6)	0.95
45 s after propofol		95 ± 18	-28 (-35, -22)	<0.001**
90 s after rocuronium		90 ± 18	-33 (-40, -27)	<0.001**
60 s after intubation	1	118 ± 25	-5 (-12, 1)	0.036
180 s after intubation	1	104 ± 24	-19 (-25, -12)	<0.001**
**Diastolic blood pressure, mmHg**				
Before start of induction		80 ± 11		
During preoxygenation with face mask		80 ± 14	-0.4 (-5, 4)	0.76
45 s after sufentanil		80 ± 13	-0.8 (-5, 4)	0.61
45 s after propofol		62 ± 14	-19 (-23, -14)	<0.001**
90 s after rocuronium		57 ± 13	-23 (-28, -18)	<0.001**
60 s after intubation	1	79 ± 17	-1 (-6, 3)	0.48
180 s after intubation	1	68 ± 18	-13 (-17, -8)	<0.001**
**Systemic vascular resistance index, dyn*s*cm^{-5}*m^{2}**				
Before start of induction		2309 ± 544		
During preoxygenation with face mask		2166 ± 601	-143 (-290, 4)	0.001
45 s after sufentanil		2059 ± 647	-250 (-397, -103)	<0.001**
45 s after propofol		1744 ± 614	-565 (-712, -419)	<0.001**
90 s after rocuronium		1764 ± 635	-546 (-692, -399)	<0.001**
60 s after intubation	2	2311 ± 694	5 (-143, 153)	0.94
180 s after intubation	2	1995 ± 735	-310 (-458, -162)	<0.001**

**Heart rate, bpm**				
Before start of induction		72 ± 13		
During preoxygenation with face mask		73 ± 15	1 (-3, 5)	0.43
45 s after sufentanil		83 ± 20	11 (7, 16)	<0.001**
45 s after propofol		70 ± 11	-2 (-7, 2)	0.12
90 s after rocuronium		64 ± 12	-8 (-12, -3)	<0.001**
60 s after intubation	1	73 ± 15	0.6 (-4, 5)	0.71
180 s after intubation	1	70 ± 15	-2 (-6, 3)	0.31
**Stroke volume index, mL*m^{-2}**				
Before start of induction		45 ± 6		
During preoxygenation with face mask		47 ± 8	2 (1, 4)	<0.001**
45 s after sufentanil		46 ± 6	1 (-0.4, 3)	0.027
45 s after propofol		46 ± 8	2 (0.04, 3)	0.005
90 s after rocuronium		48 ± 9	3 (2, 5)	<0.001**
60 s after intubation	2	45 ± 8	0.4 (-1, 2)	0.53
180 s after intubation	2	46 ± 9	2 (-0.03, 3)	0.008
**Cardiac index, L*min^{-1}*m^{-2}**				
Before start of induction		3.2 ± 0.6		
During preoxygenation with face mask		3.4 ± 0.7	0.2 (-0.02, 0.3)	0.004
45 s after sufentanil		3.7 ± 0.9	0.5 (0.3, 0.7)	<0.001**
45 s after propofol		3.5 ± 0.8	0.2 (0.06, 0.4)	<0.001**
90 s after rocuronium		3.1 ± 0.8	-0.1 (-0.3, 0.05)	0.06
60 s after intubation	2	3.3 ± 0.6	0.02 (-0.2, 0.2)	0.80
180 s after intubation	2	3.3 ± 0.8	0.01 (-0.2, 0.2)	0.88

* From linear mixed model
** statistical significant after using Bonferroni correction (i.e., alpha = 0.05/7/6 = 0.0011)
